# A Cocoa Diet Can Partially Attenuate the Alterations in Microbiota and Mucosal Immunity Induced by a Single Session of Intensive Exercise in Rats

**DOI:** 10.3389/fnut.2022.861533

**Published:** 2022-04-11

**Authors:** Patricia Ruiz-Iglesias, Malén Massot-Cladera, Maria J. Rodríguez-Lagunas, Àngels Franch, Mariona Camps-Bossacoma, Margarida Castell, Francisco J. Pérez-Cano

**Affiliations:** ^1^Secció de Fisiologia, Departament de Bioquímica i Fisiologia, Facultat de Farmàcia i Ciències de l’Alimentació, Universitat de Barcelona (UB), Barcelona, Spain; ^2^Institut de Recerca en Nutrició i Seguretat Alimentària (INSA-UB), UB, Santa Coloma de Gramenet, Spain; ^3^Centro de Investigación Biomédica en Red de Fisiopatología de la Obesidad y la Nutrición (CIBEROBN), Instituto de Salud Carlos III, Madrid, Spain

**Keywords:** acute exercise, cocoa fiber, IgA, lymphocytes, polyphenols, SCFA, tight junctions

## Abstract

**Background:**

Following intensive sports events, a higher rate of upper respiratory tract infections and the appearance of gastrointestinal symptomatology have been reported. We aimed to evaluate the effect of a cocoa-enriched diet on the cecal microbiota and mucosal immune system of rats submitted to high-intensity acute exercise, as well as to elucidate the involvement of cocoa fiber in such effects.

**Methods:**

Wistar rats were fed either a standard diet, a diet containing 10% cocoa providing 5% fiber and a diet containing only 5% cocoa fiber. After 25 days, half of the rats of each diet performed an exhaustion running test. Sixteen hours later, samples were obtained to assess, among others, the cecal microbiota and short chain fatty acids (SCFAs) composition, mesenteric lymph nodes (MLNs) and Peyer’s patches (PPs) lymphocyte composition, and immunoglobulin (Ig) content in salivary glands.

**Results:**

The intake of cocoa, partially due to its fiber content, improved the SCFA production, prevented some changes in PPs and in MLNs lymphocyte composition and also decreased the production of proinflammatory cytokines. Cocoa diet, contrary to cocoa fiber, did not prevent the lower salivary IgM induced by exercise.

**Conclusion:**

A cocoa dietary intake can partially attenuate the alterations in microbiota and mucosal immunity induced by a single session of intensive exercise.

## Background

Nutrition is important to meet the body’s energy and structural needs, as well as to improve the functionality of various body systems. Among these, immune function can be improved by several foods (1). On the other hand, although everybody is aware of the healthy influence of regular moderate-intensity training, physical exercise is often confined to one-time exercise bouts with no prior adequate training. In fact, participation in running races has become increasingly popular during recent decades. Following intensive sports events, a higher rate of mucosal infections, such as upper respiratory tract infections (URTIs) (2), and the appearance of gastrointestinal symptomatology have been reported (3, 4). A single bout of intense exercise is able to alter the counts of circulating leukocytes (5), especially T lymphocytes, probably due to both a redistribution within lymphoid and non-lymphoid organs (6) and an increased apoptosis of highly differentiated T cells (7). While the spleen seems to act as the main donor organ of T lymphocytes in the redistribution induced by exercise, Peyer’s patches (PPs) are one of the main receiving compartments (6, 8). T lymphocyte function can also be impaired following acute exercise, for instance a decreased proliferation ability (9) and a reduced type 1 T helper (Th1) cell cytokine production have been reported (10). Changes in salivary immunoglobulin (Ig) A, as a marker of mucosal immunity, have been widely studied in the field of exercise immunology (10). Although most of the studies point to a decrease in salivary IgA following intense chronic exercise (11), there is no consensus on the impact of acute exercise on this variable (12). Moreover, acute exercise alters intestinal motility, villi structure (13) and increases the gut permeability (14), probably through the alteration of tight junction (TJ) protein expression, redistribution or phosphorylation state (15). Exercise also modifies the intestinal microbiota composition and function (13, 16), as well as its crosstalk with the host, since, among other changes, a decrease in the cell-surface toll like receptor (TLR) expression has also been described (17).

Cocoa consumption has shown antioxidant (18), anti-inflammatory (19) and immunomodulatory (20) properties. These effects are mostly attributed to cocoa flavonoids, mainly (-)-epicatechin, catechin and procyanidins (21), although cocoa also contains other bioactive compounds such as theobromine and a high proportion of fiber (22). Clinical evidence suggests that cocoa and its flavonoids may be useful for the nutritional management of cardiovascular diseases, obesity and diabetes, among other chronic diseases (23), as well as for the prevention of allergies (24). Moreover, although cocoa does not show a clear ergogenic effect in humans, unlike other polyphenol sources (25), its regular intake can be helpful to avoid the intensive exercise-induced oxidative stress (18). On the other hand, preclinical studies have demonstrated cocoa’s ability to modulate both systemic and intestinal immune function (20). In this regard, cocoa intake in rats favors the Th1 response, modulates the mucosal IgA secretion and induces beneficial changes in the composition and function of the gut microbiota, for instance increasing the production of short chain fatty acids (SCFAs) and inducing a differential pattern of TLR gene expression (20, 22, 26). In addition, polymeric cocoa polyphenols are not absorbed in the small intestine and are metabolized in the colon by the gut microbiota, generating secondary bioactive metabolites that are then bioavailable and may exert even greater effects than the original polyphenols (27). Both the cocoa polyphenols and their secondary metabolites can modulate the microbiota composition and function (22, 27). This, together with the high percentage of dietary fiber present in cocoa, can explain the prebiotic properties that have been attributed to cocoa (22, 27).

Considering this background, we hypothesize that cocoa intake can prevent the intestinal and immune alterations induced by a single intense exercise bout. Hence, we aimed to evaluate the effect of a cocoa-enriched diet on the physical performance, the cecal microbiota and the mucosal immune system of rats submitted to high-intensity acute exercise, as well as to elucidate the involvement of cocoa fiber in such effects.

## Materials and Methods

### Animals

Forty-eight female Wistar rats (4-week-old) were obtained from Envigo (Huntingdon, United Kingdom) and maintained in polycarbonate cages (4 rats per cage) in the animal facilities of the Faculty of Pharmacy and Food Science at the University of Barcelona. Female rats were used because previous studies (28–30) showed a better adaptation to the treadmill in female than in male rats, while the immunological variables assessed were not modified by gender (28, 29). Animals were kept under conditions of controlled temperature and humidity in a 12:12 light-dark cycle and had access to food and water *ad libitum*. Animal handling was carried out on a scheduled basis (from 9:00 a.m. to 12:00 p.m.) to avoid the influence of biological rhythms. Rat body weight and the intake of food and water per cage were monitored throughout the study. The animal procedure was approved by both the Ethical Committee for Animal Experimentation of the University of Barcelona and the Catalonia Government (CEEA/UB ref. 517/18 and DAAM 9257, respectively).

### Diets and Acute Exercise Protocol

The acute exercise protocol applied was based on the one reported by Chaves et al. (13), with some adaptions. Firstly, to avoid biased distribution of animals, and before starting experimental diets, all rats were adapted to a specialized treadmill (Exer3/6, Columbus, OH, United States) for 1 week by increasing progressively both the running time and the treadmill speed (starting with speed 0). Then, the animals were subjected to a preselection exhaustion test (ET), which consisted of running 10 min at 18 m/min, and from then on, increasing the speed 3 m/min every 2 min until exhaustion, which was defined as the time that rats remained at the shock grid or they touched it more than three times. After checking the ability to run in the preselection ET, rats were homogeneously distributed into 6 groups (*n* = 8/each): REF/C, REF/R, C10/C, C10/R, CF/C, and CF/R that will receive three different diets. C (i.e., control) groups refer to the animals that did not run a final bout of ET (see later), and R (i.e., runner) groups refer to those that ran a final bout of ET. The REF groups (i.e., REF/C and REF/R groups) were fed with the standard diet AIN-93M (maintenance diet from the American Institute of Nutrition, Envigo). AIN-93M diet was selected as a reference diet and therefore as a base for the supplemented experimental diets in order to perform the same dietary intervention that was used in previous studies in animals with similar age (26, 31). The C10 groups (i.e., C10/C and C10/R groups) received a special chow with 10% defatted cocoa (Idilia Foods S.L., Barcelona, Spain). The CF groups (i.e., CF/C and CF/R groups) were fed with a diet containing 5% cocoa fiber powder (Idilia Foods S.L.), which provided a similar fiber cocoa content as the C10 diet but with a lower amount of polyphenols (Table 1), as applied in previous studies (32). The experimental chows used in the C10 and CF groups were elaborated on the basis of the AIN-93M formula by subtracting the amount of carbohydrates, proteins, lipids and insoluble fiber provided by the cocoa and cocoa fiber powders, resulting being isoenergetic to the REF diet (Table 1).

**TABLE 1:** Composition of the experimental diets (g/kg diet).

Components	Reference diet (g/kg)	10% Cocoa diet (g/kg)	5% Cocoa fiber diet (g/kg)
Proteins	141.8	142.6	141.1
Carbohydrates	720.7	697.9	705.1
Lipids	40.0	40.1	38.0
Insoluble fiber	50.0	54.0	56.0
Soluble fiber	0	6.0	8.0
Polyphenols	0	3.6	0.4
Theobromine	0	1.8	0.6
Other (minerals, vitamins,…)	47.5	54.0	50.9

Nutritional intervention lasted for 25 days. In the last week all rats were again adapted to run on the treadmill for 1 week. Afterward, R groups (REF/R, C10/R and CF/R groups) performed a final ET on a treadmill, while C groups (REF/C, C10/C and CF/C groups) did not. The final bout of ET was carried out in the afternoon, between 5:00 and 8:00 p.m., and consisted of running 15 min at 18 m/min, and from then on, increasing the speed 3 m/min every 2 min until exhaustion. The run length was recorded for each rat. The animals were weighted before and after performing the ET to assess the body weight loss. Moreover, all the feces made during the ET were collected and weighted both fresh and after drying, to assess their water content. Simultaneously, feces from the control rats were obtained and weighted in similar conditions with no exercise.

### Sample Collection and Processing

Sixteen hours after performing the final ET in order to avoid immediate, transitory effects (in the following day, between 9:00 a.m. and 12:00 p.m.) and without food deprivation, animals were anesthetized intramuscularly with 90 mg/kg of ketamine (Merial Laboratories S.A. Barcelona, Spain) and 10 mg/kg of xylazine (Bayer A.G., Leverkusen, Germany) and exsanguinated. Urine, feces, mesenteric lymph nodes (MLNs), small intestine, cecal content (CC) and submaxillary salivary glands (SMGs) were collected.

The small intestine was weighted and then a 0.5 cm piece (20–30 mg of tissue) of its middle part was immediately immersed in RNAlater® (Ambion, Life Technologies, Madrid, Spain), kept at 4°C overnight and then stored at –20°C until the RNA extraction. The rest of the small intestine was flushed with cold phosphate buffered saline, pH 7.2 (PBS), to remove its content. Then the proximal part of the small intestine was cut into small pieces, weighed, and incubated in shaking with 4 mL of PBS for 10 min at 37°C (55 shakes/min). After centrifugation (538 *g*, 4°C, 10 min), supernatants (gut wash, GW) were collected and stored at –20°C until IgA quantification (33). On the other hand, the distal half of the small intestine was opened lengthwise in order to collect the visible Peyer’s patches (PPs).

The lymphocytes from MLNs (MLNLs) and PPs (PPLs) were isolated by mechanically crushing the tissues through a sterile mesh cell strainer (Thermo Fisher Scientific, Barcelona, Spain) (34). PPs were previously incubated in shaking with 1 mM dithiothreitol (DTT) for 5 min at 37°C (55 shakes/min). The number and viability of the obtained lymphocytes were determined by a Countess Automated Cell Counter (Invitrogen, Barcelona, Spain).

SMGs, CC, and feces were homogenized using a tissue homogenizer (Polytron, Kinematica, Lucerne, Switzerland, for SMG; Pellet Pestle Cordless Motor, Kimble, Meiningen, Germany, for CC and feces), as previously detailed (33). After centrifugation (538 *g* for SMGs and 400 *g* for CC and feces, 4°C, 5 min), the supernatants were collected and stored at –20°C until Ig quantification.

### Urine Total Phenolic Content Quantification

The Folin-Ciocalteu method was used to determine the total phenolic content in urine, as previously described (34). Absorbance was quantified on a microplate photometer (Sunrise™, Tecan, Männedorf, Switzerland) and data were interpolated using a gallic acid standard curve (0–32 μg/mL). The polyphenol content was then normalized by urine creatinine concentration, which was measured using the Creatinine Urinary Detection Kit (Invitrogen) according to the manufacturer’s instructions.

### Immunoglobulin A-Coated Bacteria Proportion

The proportion of IgA-coated bacteria (IgA-CB) was evaluated as an indirect immune biomarker of the intestinal immune response against pathogens (35). IgA-CB in CC was determined by flow cytometry as previously described (26), with minor modifications. In the current study, the CC homogenate was stained with rabbit anti-rat Ig polyclonal antibody conjugated to fluorescein isothiocyanate (FITC) (Abcam, Cambridge, United Kingdom). Bacteria were gated in a FacsAria SORP sorter (BD Biosciences, Madrid, Spain), after propidium iodide (PI) staining (Sigma-Aldrich, Madrid, Spain) and according to their forward (FSC) and side scatter (SSC) characteristics. Analysis was performed in the Flow Cytometry Unit (FCU) of the Scientific and Technological Centers of the University of Barcelona (CCiTUB) using the FlowJo v.10 software (Tree Star, Inc., Ashland, Oregon, United States).

### Microbiota Analysis by Fluorescence *in situ* Hybridization Coupled to Flow Cytometry

Two of the most studied bacterial groups involved in the microbiota changes induced by exercise, the *Clostridium coccoides/Eubacterium rectale* and *Lactobacillus/Enterococcus* were determined by fluorescence *in situ* hybridization coupled to flow cytometry (FISH-FCM) analysis using group-specific fluorochrome-conjugated probes (Erec482 5′-GCTTCTTAGTCARGTACCG and Lab158 5′-GGTATTAGCAYCTGTTTCCA, respectively) (Sigma-Aldrich), which hybridize the bacterial 16S RNA of each particular group as previously detailed (26). Data were acquired by a FacsAria SORP sorter (BD Biosciences) in the FCU of the CCiTUB and the analysis was performed with FlowJo v.10 software (Tree Star). The results were normalized by total bacteria, which was detected adding PI (Sigma-Aldrich) prior to the flow cytometry acquisition. Commercial Flow Check™ Fluorospheres (Beckman Coulter, Inc., FL, United States) were used to assess the total counts of bacteria.

### Short Chain Fatty Acid Analysis

Acetic, propionic, butyric, valeric, and isobutyric acids were quantified in CC homogenates by headspace gas chromatography-mass spectrometry (HSGC-MS) at the GC-MS Unit of the CCiTUB, as previously detailed [31]. A 10 mM volatile free acid mix serial dilution (Supelco, Bellefonte, PA, United States) containing acetic, propionic, butyric, valeric and isobutyric acids was used as standard. The lower limits of detection were 0.404 μmol/g for acetic acid, 0.068 μmol/g for propionic acid, 0.020 μmol/g for butyric acid, 0.001 μmol/g for valeric acid, and 0.003 μmol/g for isobutyric acid.

### Immunoglobulin Quantification

The concentration of IgA and/or IgM in SMG, GW, CC, and feces were determined by a sandwich ELISA (Bethyl Laboratories Inc., Montgomery, TX, United States), as previously explained (36). Absorbance was quantified on a microplate photometer (Labsystem Multiskan, Helsinki, Finland) and data were interpolated by Ascent v.2.6 software (Thermo Fisher Scientific) according to the respective standard curves. The Ig content in SMG, CC and feces was normalized by the total protein concentration, which was determined using the Pierce-660 nm ready-to-use Protein Assay Reagent (Thermo Fisher Scientific) following the manufacturer’s instructions.

### Peyer’s Patches and Mesenteric Lymph Nodes Lymphocyte Phenotypic Analysis

MLNLs and PPLs were extracellularly stained with mouse anti-rat monoclonal antibodies (mAb) conjugated to either FITC, phycoerythrin (PE), peridinin-chlorophylla protein (PerCP), allophycocyanin (APC) and brilliant-violet 421 (BV421), as previously detailed (37). The fluorochrome-conjugated mAb used were targeted to TCRαβ, CD4, CD8α, CD8β, NKR-P1A (CD161), TCRγδ, and CD45RA (BD Biosciences). A negative control staining without any mAb and a staining control for each mAb were included. Data were acquired by Gallios Cytometer (Beckman Coulter, Miami, FL, United States) in the FCU of the CCiTUB and the analysis was performed with FlowJo v.10 software (Tree Star). Results are expressed as the proportion of positive cells in the lymphocyte population selected according to their FSC/SSC characteristics or the positive cells in a particular lymphocyte subset.

### Mesenteric Lymph Nodes Lymphocyte Stimulation and Proliferation

MLNLs (10^5^ cells/well) were incubated in 96-well plates (TPP, Sigma-Aldrich) and stimulated or not with concanavalin A (ConA, 5 μg/mL, Sigma-Aldrich). After 48 h, supernatants were collected and stored at –80°C until cytokine production analysis while the MLNLs proliferation capacity was quantified by BrdU Cell Proliferation Assay (Roche, Madrid, Spain). The proliferation rate was obtained by dividing the absorbance of ConA-stimulated cells with that of non-stimulated cells. Results are represented as fold change in the proliferation rate, considering the mean of REF/C group values as 1.

### Cytokine Quantification

Interferon (IFN)-γ, interleukin (IL)-2, IL-10, IL-4, IL-6, and tumor necrosis factor (TNF)-α production was determined in ConA-stimulated MLNLs supernatants by ProcartaPlex Multiplex Immunoassay (Affymetrix, eBioscience, San Diego, United States), according to the manufacturer’s protocol. Data were acquired by MAGPIX Cytometer (Affymetrix) in the FCU of the CCiT-UB and analyzed by ProcartaPlex Analyst v1.0 software (Affymetrix). The lowest limits of detection were 3.34 pg/mL for IFN-γ, 1.82 pg/mL for IL-2, 6.01 pg/mL for IL-10, 0.62 pg/mL for IL-4, 2.19 pg/mL for IL-6, and 2.88 pg/mL for TNF-α.

### Quantification of Gene Expression in Small Intestine

Polymeric Ig receptor (pIgR), retinoic acid receptor (RAR)-α, forkhead box P3 (FoxP3), TLR2, TLR4, TLR5, TLR7, free fatty acid receptor (FFAR) 2, FFAR3, occludin (Ocldn), claudin-4 (Cldn4), zonula occludens (ZO)1, and ZO2 gene expression were assessed in the small intestine, as previously described (37). Briefly, the small intestine portion kept in RNA later was homogenized in a lysing matrix tube (MP Biomedicals, Illkirch, France) by a FastPrep-24 instrument (MP Biomedicals) for 30 s and the RNA contained was isolated with the RNeasy mini kit (Qiagen, Madrid, Spain). RNA was quantified using a NanoPhotometer (BioNova Scientific S.L., Fremont, CA, United States) and reverse-transcribed using TaqMan Reverse Transcription Reagents (Applied Biosystems, AB, Weiterstadt, Germany). Afterward, the Real Time (RT)-PCR was carried out using the ABI Prism 7900 HT quantitative RT-PCR system (AB) and the following specific PCR TaqMan primers (AB): pIgR (Rn00562362_m1, Inventoried, I), RARα (Rn00580551_m1, I), FoxP3 (Rn01525092_m1, I), TLR2 (Rn02133647_s1, I), TLR4 (Rn00569848_m1, I), TLR5 (Rn04219239_s1, I), TLR7 (Rn01771083_m1, I), FFAR2 (Rn02345824_s1, I), FFAR3 (Rn01457614_g1, I), Ocldn (Rn00580064_m1, I), Cldn4 (Rn01196224_s1, I), ZO1 (Rn02116071_s1, I) and ZO2 (Rn01501483_m1, I). Data was analyzed with SDS version 2.4 software (AB). The housekeeping genes Gusb (β-glucuronidase, Rn00566655_m1, I) and β-actin (Rn00667869_m1, I) were used for normalizing the gene expression of the target genes, using the 2^–ΔΔ*Ct*^ method (38). Since the target and the housekeeping genes should have a similar expression level, the gene expression of β-actin was used as housekeeping gene for pIgR, RARα, TLR5, Ocldn, Cldn4, and ZO2, whereas Gusb expression was used for the rest of the genes. Fold changes in small intestine gene expression are expressed considering the mean of REF-C group values as 1.

### Statistical Analysis

Statistical analysis of the data was performed using IBM Social Sciences Software Program (SPSS, version 26.0, Chicago, IL, United States). Normality and variance equality of the data were confirmed by Shapiro-Wilk’s and Levene’s test, respectively. A two-way ANOVA test was applied and, if significant differences were detected, Tukey’s *post-hoc* test was carried out. When results were neither equally nor normally distributed, a Kruskal-Wallis test was used and, if significant differences were detected, Dunnet’s test was performed as a *post-hoc* test. When comparing variables throughout the study (e.g., body weight, chow intake and water intake), the repeated measures ANOVA test was used to assess whether there was a significant interaction between time (day of study) and the dietary condition (REF, CF and C10 diets). Once we confirmed there was a significant interaction, a one-way ANOVA test followed by the Tuckey’s *post-hoc* test was used to detect between which days of study the differences between the dietary groups were statistically significant. Significant differences were considered when *p* < 0.05.

## Results

### Body Weight, Chow Intake, Urine Flavonoid Content, and Exercise Performance

**FIGURE 1:**
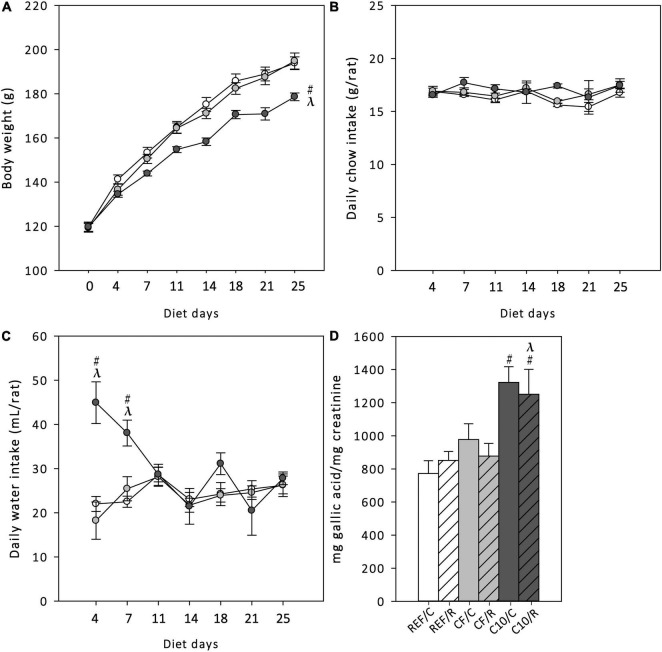
Body weight **(A)**, chow intake **(B)** and water intake **(C)** throughout the 25-day dietary intervention period, and urine total polyphenol content at the end of the study **(D)**. REF, reference diet; CF, 5% cocoa fiber-enriched diet; C10, 10% cocoa-enriched diet. The REF groups are represented by white symbols (○) or white bars, the CF groups by gray symbols (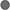) or gray bars and the C10 by black symbols (●) or black bars. In **(D)**, the sedentary control groups (C) are represented by smooth bars and the runner groups (R) by striped bars. Data are expressed as mean ± SEM (*n* = 8). Statistical differences [repeated measures ANOVA test followed for **(A–C)** and Kruskal-Wallis followed by Dunnet’s test for **(D)**]: ^#^*p* < 0.05 vs. REF diet; ^λ^*p* < 0.05 vs. CF diet.

Body weight (BW), chow and water intake were monitored every 3–4 days throughout the 25-days nutritional intervention (Figure 1). The BW increased progressively during the study period in all dietary groups (time effect *p* = 0.001 by repeated-measures ANOVA), but the animals that received the C10 diet had a lower BW gain than the REF and CF groups (Figure 1A) (Time x Diet interaction effect *p* = 0.001 by repeated-measures ANOVA), while they consumed a similar amount of chow than REF animals (Figure 1B). The water intake was much higher in the C10 group during the first week of nutritional intervention (Figure 1C).

To confirm the intestinal absorption of the polyphenols contained in cocoa diets, the total polyphenol content in urine was quantified at the end of the study in the six groups of animals (Figure 1D). The C10 diet (both C10/C and C10/R groups), with the highest content of polyphenols, significantly increased the total urine polyphenol content.

**FIGURE 2:**
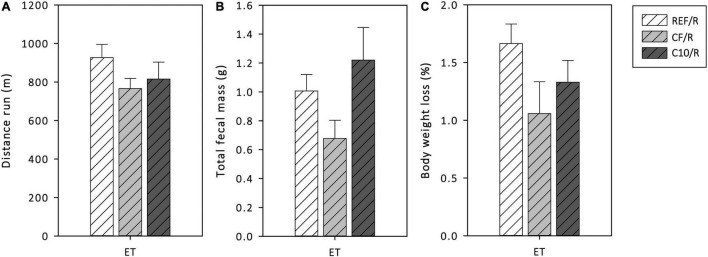
Distance run **(A)**, total fecal mass defecated **(B)** and body weight loss **(C)** during the final exhaustion test (ET). REF, reference diet; CF, 5% cocoa fiber-enriched diet; C10, 10% cocoa-enriched diet; R, runner groups. Data are expressed as mean ± SEM (*n* = 8).

During the final ET, the distance run, the total fecal mass produced, and the BW loss were monitored (Figure 2). The REF animals ran more than 900 m, produced about 1 g of feces and lost about 1.7 g of BW during the final ET. Neither C10 nor CF diets significantly modified these variables. However, the CF enriched diet tended to decrease the total fecal mass and the BW loss compared to the other runner groups (*p* < 0.1).

### Fecal Water Content, Cecal pH, and Small Intestine Weight

The influence of a single bout of intensive exercise and the cocoa-enriched diets on fecal humidity (water content), cecal pH and small intestine weight were also assessed at the end of the study (Table 1). Feces during intensive exercise had a higher water content but the CF diet—but not C10 diet—avoided this increase. There were no changes in the cecal pH (Table 2).

**TABLE 2:** Water content of feces defecated during the exhaustion test and the same time in the control rats, cecal content pH, and small intestine weight at the end of the study.

	Group	REF	CF	C10
Fecal water content (%)	C	46.12 ± 0.94	48.91 ± 1.21	46.68 ± 0.12
	R	53.68 ± 3.07*	49.96 ± 1.72	53.13 ± 2.34*
Cecal content pH	C	7.63 ± 0.09	7.65 ± 0.11	7.57 ± 0.11
	R	7.65 ± 0.09	7.54 ± 0.12	7.78 ± 0.10
Small intestine weight (g/100 g BW)	C	100.00 ± 2.17	88.46 ± 3.21^#^	108.66 ± 2.60^#λ^
	R	94.40 ± 2.80	92.55 ± 2.09^#^	110.29 ± 2.46^#λ^

REF, reference diet; CF, 5% cocoa fiber enriched diet; C10, 10% cocoa enriched diet. Data are expressed as mean ± SEM (n = 8). C, control group; R, runner group. Statistical differences (Kruskal-Wallis followed by Dunnet’s test): *p < 0.05 vs. the C group in the same diet; ^#^p < 0.05 vs. the same exercise condition in the REF diet; ^λ^p < 0.05 vs. the same exercise condition in the CF diet.

Exercise did not modify the small intestine weight, independently of the dietary condition (Table 2). However, following the C10 diet, the small intestine weight rose.

### Cecal Microbiota Composition

**FIGURE 3:**
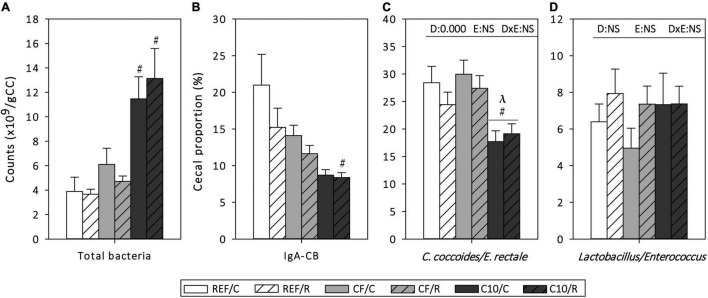
Cecal microbiota composition: counts of total bacteria **(A)**, proportion of IgA-coated bacteria **(B)**, and proportions of *Clostridium coccoides/Eubacterium rectale* **(C)** and *Lactobacillus/Enterococcus* **(D)** in cecal content homogenates. The control (C) groups are represented by smooth bars and the runner (R) groups by striped bars. REF, reference diet; CF, 5% cocoa fiber-enriched diet; C10, 10% cocoa-enriched diet; NS, no statistically significant differences detected. Data are expressed as mean ± SEM (*n* = 8). Statistical differences (Kruskal-Wallis followed by Dunnet’s test): ^#^*p* < 0.05 vs. the same exercise condition in the REF diet; ^λ^*p* < 0.05 vs. the same exercise condition in the CF diet. The inset table shows two-way ANOVA results when applied (D, diet; E, exercise, DxE, interaction between diet and exercise).

The amount of cecal bacteria, the proportion of them coated to IgA (IgA-CB), as well as the cecal proportion of *Clostridium coccoides/Eubacterium rectale* and *Lactobacillus/Enterococcus* in CC were established in samples obtained 16 h after the final exhaustion (Figure 3). Acute exercise did not modify these variables. However, the consumption of the C10 diet, but not that of CF, increased, by more than threefold, the amount of total bacteria in the cecum in both exercised conditions (*p* < 0.01). Moreover, C10 diet resulted in a lower proportion of IgA-CB in runner rats (*p* < 0.05). C10 consumption, but not that of CF, decreased the proportion of *C. coccoides/E. rectale* in both exercise conditions (*p* < 0.01) without modifying that of *Lactobacillus/Enterococcus.*

### Cecal Short Chain Fatty Acids Production

**FIGURE 4:**
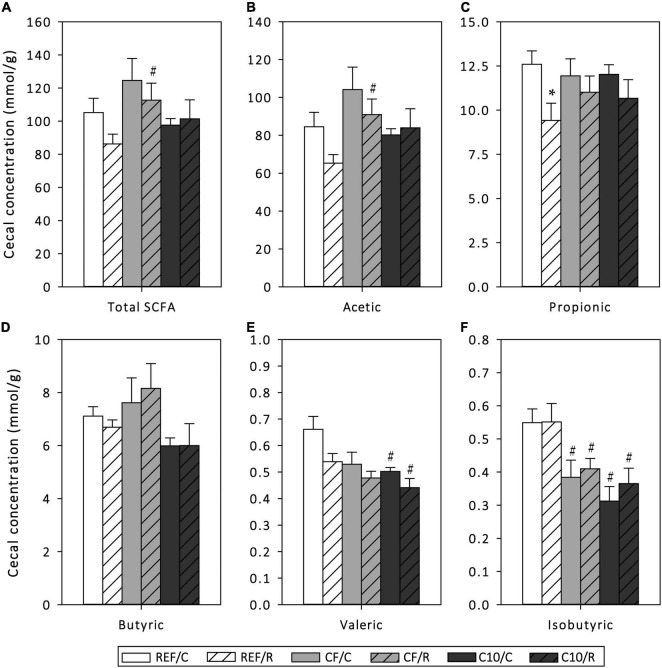
Cecal short-chain fatty acid (SCFA) production: amount of total SCFAs **(A)**, acetic acid **(B)**, propionic acid **(C)**, butyric acid **(D)**, valeric acid **(E)** and isobutyric acid **(F)** in cecal content homogenates. The control (C) groups are represented by smooth bars and the runner (R) groups by striped bars. REF, reference diet; CF, 5% cocoa fiber-enriched diet; C10, 10% cocoa-enriched diet. Data are expressed as mean ± SEM (*n* = 8). Statistical differences (Kruskal-Wallis followed by Dunnet’s test): *p < 0.05 vs. the C group in the same diet; ^#^*p* < 0.05 vs. the same exercise condition in the REF diet.

The function of the gut microbiota was assessed by means of cecal SCFA quantification (Figure 4). Overall, acute exercise significantly decreased the production of propionic (*p* < 0.05) and tended to lower that of valeric acid (*p* < 0.1). On the other hand, runner CF rats, but not C10 rats, showed a higher total SCFA concentration in cecum content than REF rats, which was mainly due to an increase in acetic acid production, the most abundant SCFA. Both CF and C10 diets prevented the decrease in propionic acid induced by exercise, although these diets did not avoid the changes in valeric acid concentration. Neither acute exercise nor the experimental diets modified the production of butyric acid. In addition, CF and C10 diets decreased the amount of isobutyric acid in sedentary control and runner rats (*p* < 0.05).

### Mucosal Immunoglobulins

**FIGURE 5:**
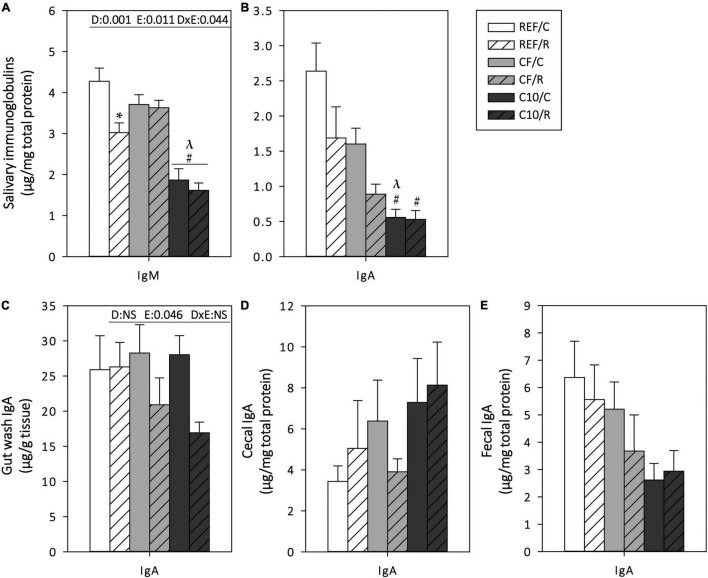
Mucosal immunoglobulins: submaxillary salivary gland IgM **(A)** and IgA **(B)**, gut wash **(C)**, cecal **(D)** and fecal **(E)** IgA contents. The control (C) groups are represented by smooth bars and the runner (R) groups by striped bars. REF, reference diet; CF, 5% cocoa fiber-enriched diet; C10, 10% cocoa-enriched diet; NS, no statistically significant differences detected. Data are expressed as mean ± SEM (*n* = 8). Statistical differences (two-way ANOVA followed by *post hoc* Tukey for **(A,C)** and Kruskal-Wallis followed by Dunnet’s test for **(B,D,E)**: *p < 0.05 vs. the sedentary group in the same dietary group; ^#^*p* < 0.05 vs. the same exercise condition in the REF diet; ^λ^*p* < 0.05 vs. the same exercise condition in the CF diet. The inset table shows two-way ANOVA results when applied (D, diet; E, exercise, DxE, interaction between diet and exercise).

Mucosal Ig concentrations were determined in SMG, GW, CC, and feces, 16 h after the final ET (Figure 5). In SMG, acute exercise induced a 30% decrease in IgM content (*p* = 0.01) and tended to decrease that of IgA (Figures 5A,B). CF diet intake prevented the decrease in IgM levels. The C10 diet decreased the IgM and IgA content in SMG in both exercise conditions.

Regarding the intestinal IgA content, the statistical analysis showed an overall decrease in GW IgA levels due to acute exercise, although these changes were only observed in CF and C10 runner groups (Figure 5C). Neither exercise nor the diets significantly modified cecal and fecal IgA (Figures 5D,E).

### Peyer’s Patches Lymphocyte Composition

**FIGURE 6:**
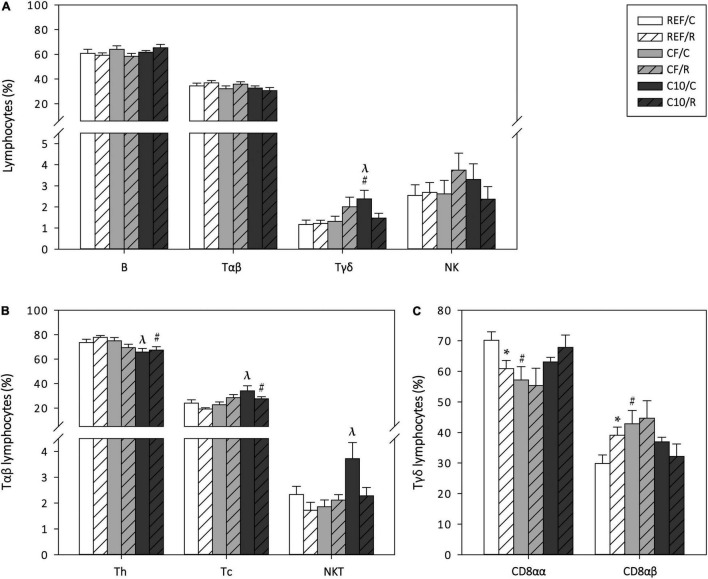
Peyer‘s patches’ lymphocyte composition: main lymphocyte subsets **(A)**, Tαβ cell subsets **(B)** and Tγδ cell subsets **(C)** cell proportion. The control (C) groups are represented by smooth bars and the runner (R) groups by striped bars. REF, reference diet; CF, 5% cocoa fiber-enriched diet; C10, 10% cocoa-enriched diet. Data are expressed as mean ± SEM (*n* = 8). Statistical differences (Kruskal-Wallis followed by Dunnet’s test): *p < 0.05 vs. the C group in the same diet; ^#^*p* < 0.05 vs. the same exercise condition in the REF diet; ^λ^*p* < 0.05 vs. the same exercise condition in the CF diet.

To assess the influence of a single bout of exercise and the experimental diets on the gut-associated lymphoid tissue, the proportion of the main lymphocyte subsets in PPs was also established 16 h after performing the final ET (Figure 6). The proportion of B cells, Tαβ cells and NK cells was not affected by acute exercise or diet. However, the C10 diet, but not the CF diet, increased the proportion of Tγδ cells in sedentary control animals (*p* < 0.05 vs. REF/C and CF/C groups). Concerning the Tαβ cell subsets, there were no changes due to acute exercise but the C10 diet decreased the Th cell proportion and reciprocally increased that of Tc cells in runner animals (*p* < 0.05). Moreover, the C10 diet, but not the CF diet, increased the proportion of NKT cells in sedentary control animals (*p* < 0.05). Regarding the Tγδ CD8αα and CD8αβ cell subsets, runner rats from the REF group showed a lower proportion of Tγδ CD8αα cells and, reciprocally, a higher one of Tγδ CD8αβ cells than sedentary counterparts (*p* < 0.05, REF/C group vs. REF/R group). In this case, the CF diet, but not the C10 diet, induced the same changes as acute exercise (*p* < 0.05, REF/C group vs. CF/C group).

### Mesenteric Lymph Nodes Lymphocyte Composition

**FIGURE 7:**
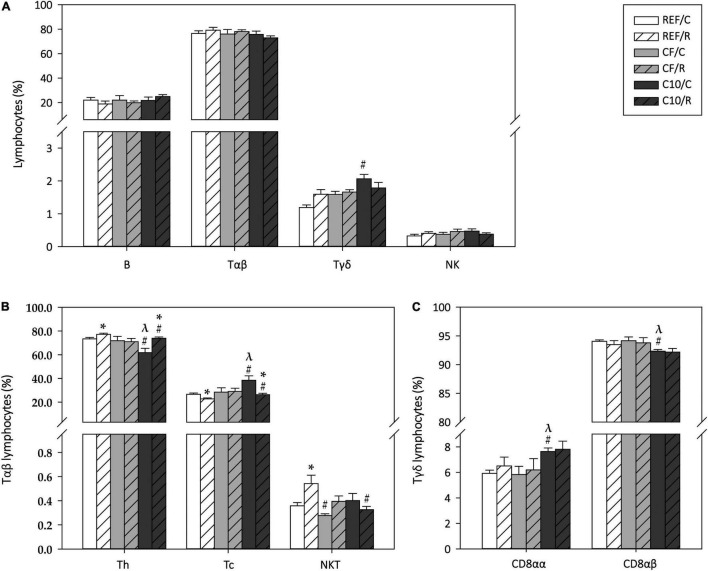
Mesenteric lymph node lymphocytes composition: proportion of main lymphocyte subsets **(A)**, Tαβ cell subsets **(B)** and Tγδ cell subsets **(C)**. The control (C) groups are represented by smooth bars and the runner (R) groups by striped bars. REF, reference diet; CF, 5% cocoa fiber-enriched diet; C10, 10% cocoa-enriched diet. Data are expressed as mean ± SEM (*n* = 8). Statistical differences (Kruskal-Wallis followed by Dunnet’s test): *p < 0.05 vs. the C group in the same diet; ^#^*p* < 0.05 vs. the same exercise condition in the REF diet; ^λ^*p* < 0.05 vs. the same exercise condition in the CF diet.

The phenotypic composition of MLNLs was also characterized 16 h after performing the final ET (Figure 7). The acute exercise did not significantly modify the proportion of B cells, Tαβ cells, Tγδ cells, and NK cells (Figure 7A). However, the C10 diet increased the proportion of Tγδ cells in the sedentary animals (*p* < 0.05).

Regarding the main Tαβ cell subsets, acute exercise increased the proportions of Th cells and NKT cells (*p* < 0.05 in both cases), with a reciprocal decrease in Tc cell percentage (*p* < 0.05 REF/R vs. REF/C) (Figure 7B). The animals fed with the C10 diet showed a lower proportion of Th cells and a higher one of Tc cells than REF and CF rats (*p* < 0.05), and the changes induced by acute exercise were also observed in this dietary group. On the other hand, the CF diet intake prevented the exercise-induced changes in Th and Tc cell proportions while both cocoa-enriched diets attenuated the change in NKT cell proportion. With regard to the Tγδ CD8αα and CD8αβ cell subsets, while exercise did not induce any change, the C10 diet, but not the CF diet, increased the proportion of the Tγδ CD8αα cells (*p* < 0.05 C10/C vs. REF/C and CF/C) while it decreased reciprocally that of Tγδ CD8αβ cells (*p* < 0.05 C10/C vs. REF/C and CF/C) (Figure 7C).

### Mesenteric Lymph Node Lymphocyte Functionality

**FIGURE 8:**
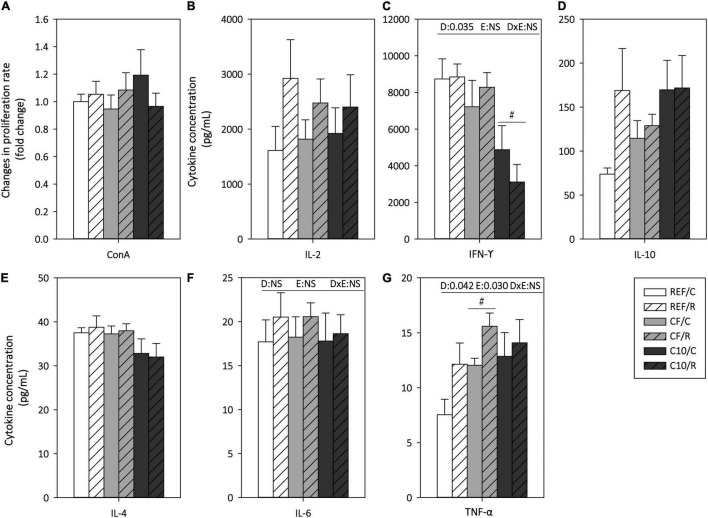
Mesenteric lymph node lymphocyte functionality: proliferation response **(A)** and cytokine concentration released under concanavalin A (Con A) stimulation **(B–G)**. The control (C) groups are represented by smooth bars and the runner (R) groups by striped bars. REF, reference diet; CF, 5% cocoa fiber-enriched diet; C10, 10% cocoa-enriched diet; NS, no statistically significant differences detected. Data are expressed as mean ± SEM (*n* = 8). Statistical differences (two-way ANOVA followed by *post hoc* Tukey for **(C,F,G)** and Kruskal-Wallis followed by Dunnet’s test for **(A,B,D,E)**: ^#^*p* < 0.05 vs. the same exercise condition in the REF diet. The inset table shows two-way ANOVA results when applied (D, diet; E, exercise, DxE, interaction between diet and exercise).

The functionality of MLN T lymphocytes was assessed by means of their proliferative response and cytokine production ability after ConA stimulation (Figure 8). Neither exercise nor the experimental diets affected significantly the MLNLs proliferation capacity (Figure 8A).

Acute exercise increased the production of TNF-α in all dietary conditions (Figure 8G), without significantly modifying that of IL-2, IFN-γ, IL-10, IL-4, and IL-6. The C10 diet, but not the CF diet, decreased the production of the proinflammatory cytokine IFN-γ in both exercise conditions. On the other hand, the CF diet increased the TNF-α production.

### Gene Expression in Small Intestine

**FIGURE 9:**
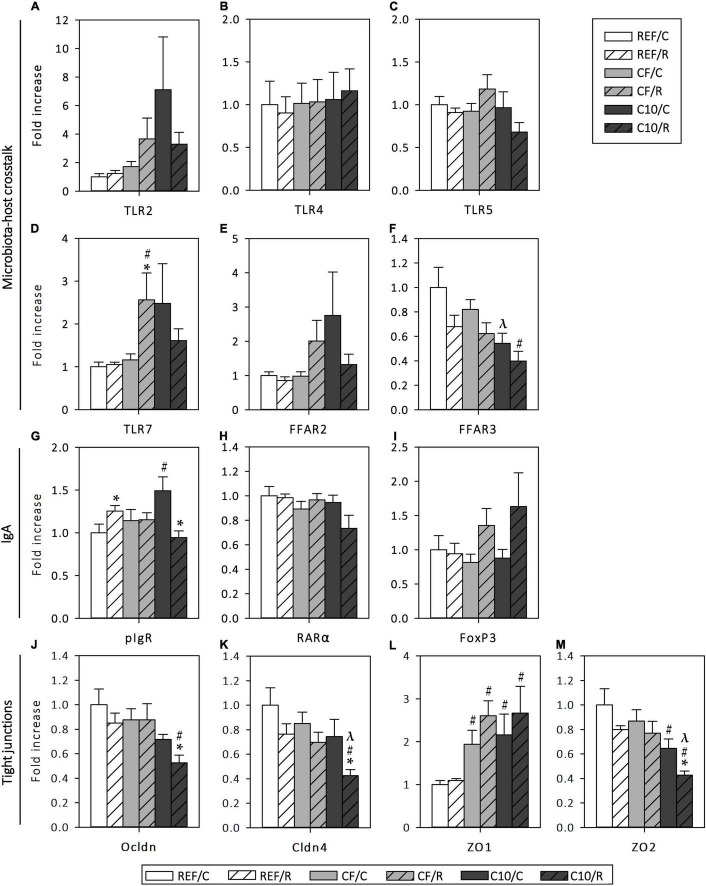
Changes in small intestine gene expression: Toll-like receptor (TLR) 2 **(A)**, TLR4 **(B)**, TLR5 **(C)**, TLR7 **(D)**, free fatty acid receptor (FFAR) 2 **(E)** and FFAR3 **(F)**, polymeric immunoglobulin receptor (pIgR) **(G)**, retinoic acid receptor (RAR) α **(H)**, forkhead box P3 (FoxP3) **(I)**; occludin (Ocldn) **(J)**, claudin-4 (Cldn4) **(K)**, *zonula occludens* (ZO) 1 **(L)** and ZO2 **(M)** gene expression. Results expressed as fold change with respect to the REF/C group. The control (C) groups are represented by smooth bars and the runner (R) groups by striped bars. REF, reference diet; CF, 5% cocoa fiber-enriched diet; C10, 10% cocoa-enriched diet. Data are expressed as mean ± SEM (*n* = 8). Statistical differences (Kruskal-Wallis followed by Dunnet’s test): *p < 0.05 vs. the C group in the same diet; ^#^*p* < 0.05 vs. the same exercise condition in the REF diet; ^λ^*p* < 0.05 vs. the same exercise condition in the CF diet.

The effect of acute exercise and the cocoa diets on the gene expression of some molecules involved in the crosstalk between the microbiota and the host, IgA production and TJ formation were also assessed in small intestine tissue (Figure 9).

A single bout of intensive exercise did not modify the gene expression of the studied TLRs (Figures 9A–D). CF runner rats, but not those fed with C10, showed a higher expression of TLR7 (*p* < 0.05, vs. CF/C and REF/R). On the other hand, C10 animals, but not CF rats, had lower levels of FFAR3 in both exercise conditions (*p* < 0.05 vs. REF and CF diets).

Regarding the small intestine gene expression of proteins involved in IgA transcytosis and IgA^+^ B cell differentiation, acute exercise in REF animals and the intake of the C10 diet increased the pIgR gene expression (*p* < 0.05 in both cases), whereas the combination of both (C10/R) unexpectedly decreased it (*p* < 0.05 vs. REF/R and C10/C) (Figure 9G). The expression of RARα and FoxP3 was not affected by either exercise or the experimental diets (Figures 9H,I).

On the other hand, a single bout of intensive exercise did not modify the gene expression of the TJ proteins Ocldn, Cldn4, ZO1 and ZO2 in REF animals (Figures 9J–M). However, both experimental diets increased by more than twofold the expression of ZO1 (*p* < 0.05 vs. REF diet) in both exercise conditions. In addition, both sedentary and runner C10 animals showed lower levels of ZO2 (*p* < 0.05), whereas the gene expression of Ocldn (*p* < 0.05, REF/R vs. C10/R) and Cldn4 (*p* < 0.05, C10/R vs. REF/R and CF/R) decreased only in C10 runner animals (*p* < 0.05 for Ocldn and Cldn4, C10/C vs. C10/R). These changes were not found in rats submitted to the CF diet.

## Discussion

In previous studies, we have demonstrated the immunomodulating (32) and antioxidant properties (31, 39) of cocoa enriched-diets, as well as the prebiotic effects (22) of cocoa fiber in both healthy rats (22, 32) and experimental models of allergy and inflammation (31, 34, 39, 40). However, there are no studies focused on these cocoa properties in a situation of excessive oxidative stress, such as in an acute intensive exercise. In the current study, we aimed to evaluate the influence of a single intense exercise bout and a 25-day cocoa-enriched diet in rats on their cecal microbiota and mucosal immune system. Likewise, we aimed to elucidate the involvement of cocoa fiber in such effects. We found that a single intense exercise bout increased the fecal humidity and, although no change in the cecal microbiota composition was observed, a reduction in some SCFAs, such as propionic acid and a trend to decrease the proportion of valeric acid was found. Moreover, a single bout of intense exercise decreased the IgM content in salivary glands and the proportion of Tγδ CD8αα cells in PPs, whereas increased the percentage of Th lymphocytes in MLNs, and raised the gene expression of the pIgR in the intestinal wall. The cocoa-enriched diet (C10), which did not modify the exercise performance or prevent the exercise-induced increased fecal humidity, was able to increase the small intestine weight and the cecal bacterial content, although it induced a lower proportion of IgA-coated bacteria and *Clostridium coccoides*/*Eubacterium rectale*, changes that could be due to other bioactive compounds rather than fiber because the CF diet, which contained the same amount of cocoa fiber and no polyphenols or methylxanthines, did not induce these changes. However, cocoa diet, due to its fiber content, avoided the decrease in cecal propionic acid but not that in valeric acid induced by exercise. Moreover, diets containing cocoa or cocoa fiber alone reduced the production of isobutyric acid. In the mucosal Igs, the cocoa diet decreased the salivary IgM and IgA contents. These changes could be explained again by the effect of other cocoa bioactive compounds rather than fiber, since the CF diet did not produce the same effect. In PPs, 25-day cocoa diet caused some changes in lymphocyte composition, partially due to its fiber content, and prevented the decrease in the proportion of Tγδ CD8αα cells, probably due to other bioactive compounds, induced by exercise. Likewise, the cocoa diet, partially because of its fiber content, reduced the proportion of Th cells but increased that of Tγδ and Tγδ CD8αα cells in MLNs, and prevented the increase in NKT cell percentage induced by exercise in this compartment. With regard to the functionality of MLNLs, reduced the production of the proinflammatory IFN-γ. Finally, after 25 days, cocoa diet, but not cocoa fiber, decreased the FFAR3 and ZO2 gene expression, whereas both diets enhanced two-to threefold that of ZO1. With regard to cocoa fiber, on its own it prevented the increase in fecal humidity, raised the production of SCFAs and the TLR7 gene expression, and avoided the decrease in salivary IgM induced by exercise.

Focusing on exercise performance, the cocoa diet did not improve it, which agrees with most of the human studies using cocoa polyphenols (25). Even so, some preclinical studies have reported that (–)-epicatechin and (–)-epicatechingallate, both flavanols present in cocoa, exert ergogenic effects in rodents (41–43), while others reported no changes (44) or even a decrease in time-to-exhaustion after a 6-month dietary intervention (45). The controversy in these results may be due to the dosage of cocoa or its polyphenol content, as well as the exercise protocol applied.

During the exhaustion test, we measured the BW loss to record the level of dehydration, as well as the total fecal mass produced and its water content, which allowed us to approach changes in gastrointestinal (GI) motility. In line with current literature (4, 46), we found that acute exercise increased fecal humidity which may be due to a faster GI motility. This fact was prevented by the intake of the cocoa fiber diet. The current consensus is to avoid a high intake of fiber 24–72 h before and during intensive exercise to prevent a consequent diarrhea (47), nevertheless, the outcome may depend on the kind of fiber, its source and the ratio of soluble and insoluble fiber consumed. Although a more specific method to evaluate changes in GI motility may be applied in future research, our results suggest that the proportion of cocoa fiber tested exerts protective effects against exercise-induced increased GI motility (48, 49). In addition, the involvement of cocoa components in the crosstalk between neurons, immune cells, and microbes, also known as the intestinal neuro-immune axis (50) also deserves to be studied in depth to ascertain the precise mechanisms involved.

In line with this and with regard to the cecal microbiota composition, acute exercise did not induce changes in the amount of total bacteria, the proportion of those bound to IgA, *Clostridium coccoides/Eubacterium rectale* and *Lactobacillus/Enterococcus* percentages or the cecal pH. Surprisingly, these results disagree with the acute exercise-induced decrease in *Lactobacillus* proportion and the increase of that of *Clostridium* found by Chaves et al. using a similar experimental design (13). Nevertheless, the available evidence is quite controversial, since other authors reported a higher *Lactobacillus* proportion in both intensively and moderately exercised rats (51, 52). Further studies may clarify the relationship established between the degree of the exercise’s intensity and its impact on gut microbiota composition in a more exhausting study. On the other hand, the intake of the cocoa-enriched diet tripled the amount of total bacteria present in cecal content, which could contribute to the higher intestinal weight found in these animals. Moreover, cocoa intake lowered the proportion of bacteria bound to IgA as well as the percentage of *Clostridium coccoides/Eubacterium rectale*, without modifying that of *Lactobacillus/Enterococcus*, results that are in line with previous studies (26). This influence of cocoa in cecal content was not due to its fiber content because the fiber enriched diet did not produce the same changes. Therefore, these changes may be attributed to the other bioactive compounds present in cocoa, such as polyphenols and methylxanthines. In line with this, we have previously reported a similar increase in the amount of total bacteria and a decrease in the proportion of them bound to IgA due to a 4-week hesperidin supplementation, which is one of the main polyphenols found in citrus fruits (53). Theobromine, the predominant xanthine found in cocoa, also seems to play an important role in lowering the proportion of IgA-coated bacteria (54). The impact of these changes depends on the kind of bacteria that was bound to IgA, since IgA can bind pathogenic bacteria to neutralize them or it can transport commensal bacteria to the intestinal epithelium. Nevertheless, it seems that IgA has a higher affinity to inflammatory bacteria (35, 55), since increased levels of this have been found in inflammatory bowel disease patients (35, 56). However, further studies may elucidate the involvement of cocoa’s polyphenols and other bioactive compounds in cocoa’s impact on cecal microbiota composition.

When assessing the impact on microbiota, it is important to take into account not only the composition but also its function. SCFAs are the main bacterial metabolites of the fermentation of dietary fiber and proteins and they exert an important role as metabolic regulators. Previous research has associated an increased gut-derived SCFA production with a better insulin sensitivity, a reduced inflammation (57) and even an improved exercise performance (58, 59), this last factor being associated with its interaction with skeletal muscle. It has been reported that the regular practice of moderate intensity exercise increases the production of butyric acid (60, 61). Moreover, some authors found an inverse correlation between the fecal concentration of propionic and acetic acids and the exercise performance in a cardiorespiratory fitness test (measured as peak oxygen uptake, VO_2_ peak) (60), whereas other studies reported higher propionic and acetic acid levels in elite professionals rugby players (61) and in lean subjects submitted to a 6-weeks endurance-based exercise intervention (62). In spite of these studies focused on long-term exercise, the impact of acute intensive exercise on SCFA production remains unclear. Our present results indicate a lower propionic acid concentration in cecum due to acute exercise, which partially agree with the findings of Estaki et al. (60). Both cocoa- and cocoa fiber- enriched diets attenuated this exercise-induced decrease in propionic acid, and even the CF groups also had higher levels of cecal acetic acid. This agrees with previous studies reporting that a 3-week diet with cocoa fiber increased the cecal concentration of acetic, propionic and butyric acids (22). A recent preclinical study pointed out the importance of acetic acid as an energy substrate during endurance exercise, since a continuous acetic acid infusion was able to restore the lost exercise capacity of mice treated with antibiotics (63). However, we did not observe a higher exercise performance linked to fiber diet. On the other hand, we also found that exercise tended to decrease the cecal content of valeric acid, which together with the branched SCFA isovaleric and isobutyric acids are considered putrefactive SCFAs (pSCFAs). These pSCFAs are products of the bacterial fermentation of proteins and are associated with the generation of other concomitant products that may be detrimental to the colonic epithelium (64). Then, the exercise-induced decrease in valeric acid might be beneficial for the host. Moreover, cocoa-enriched diet, partially because of its fiber content, induced a decrease in the concentration of valeric and isobutyric acids. These results are in line with previous studies that reported a negative correlation between dietary fiber consumption and the levels of pSCFAs (65). Some of the beneficial effects of SCFAs to the host, such as their anti-inflammatory properties, occur through the interaction with the two specific SCFAs receptors FFAR2 (GPR43) and FFAR3 (GPR41) (66, 67). Here, although the activation status of these receptors was not analyzed, a bout of intense exercise did not change the small intestine gene expression of FFAR2 and FFAR3. However, cocoa-enriched diet, but not cocoa-fiber diet, decreased the gene expression of FFAR3, which is mainly activated by propionic and butyric acids and is involved in energy balance maintenance, noradrenaline release from sympathetic neurons and the production of anorectic hormones like the peptide YY, among other functions (68). Then, these changes in the intestinal gene expression of FFAR3 may be somehow associated with the lower BW gain observed in C10 animals (and not in CF rats), here as well as in other studies from our research group (22, 33, 36) and from other authors (69, 70). Moreover, the anti-obesity potential of cocoa has already been discussed in several reviews (21, 71–73). Although we previously attributed the lower BW gain of rats fed with cocoa to the impact of theobromine and flavonoids on lipid and glucose metabolism, fat deposition and even the gut microbiota composition (22, 74), the current results suggest that a decrease in FFAR3 intestinal expression may also contribute to this multifactorial effect.

The intestinal epithelium, by means of the enterocytes, the tight junction proteins, the mucus layer and immune cells, plays an essential role as a barrier that regulates the permeability of nutrients, water and ions, and prevents the invasion of pathogenic bacteria, and together with the intestinal microbiota contributes to the maintenance of the intestinal homeostasis. Numerous studies have shown that intensive exercise impairs the expression and the phosphorylation status of the TJ proteins, disrupting the integrity of the intestinal epithelial barrier and leading to an increased gut permeability (15, 75). This impairment has been associated with the hyperthermia (76), dehydration (77) and the stress (78) induced by intensive exercise. Lambert et al. associated an increased gastric and small intestinal permeability, with a 1.5% of BW loss in trained distance runners after running without fluid for 60 min at 70% VO_2_ max (77). The level of dehydration described is like that we found in REF animals. Although we did not assess changes in intestinal permeability *per se*, we studied the effect of exercise on the small intestine gene expression of TJ proteins, which are essential for the regulation of gut paracellular permeability. The acute exercise protocol applied in the current study did not induce changes in Ocldn, Cldn4, ZO1 or ZO2 small intestine gene expression in REF animals. These results are in line with those of Chaves et al. (13), showing no changes in cldn1 and ZO1 intestinal expression of rats 12 h after performing acute exercise without prior training, although they observed a larger colonic villi interspace in runner rats. Nevertheless, Ducray et al. reported a lower ZO1 intestinal protein expression in rats 4 h after exhaustion running (79). On the other hand, cocoa diet, due to its fiber content, substantially increased the gene expression of ZO1, which agrees with studies recently published (80) and could be somehow associated with the protective effect of cocoa fiber on the increased GI motility induced by acute exercise. Taking together with the results regarding microbiota and intestinal epithelium, our results here suggest that cocoa consumption can improve intestinal health, especially by means of increasing cecal SCFA production and ZO1 expression, which could be useful not only to prevent the alterations induced by intensive exercise, but also in situations with an even more impaired intestinal barrier function, like some gastrointestinal disorders.

Whereas the study of exercise’s impact on gut microbiota has just gained importance in recent years, its effect on IgA production has been widely studied. IgA is the main Ig isotype in the mucosal compartment and plays many essential roles, such as neutralization of pathogens and toxins, antigen sampling and blocking excessive commensal and pathogenic bacteria (55). It has been reported that regular sessions of moderate intensity exercise increase salivary IgA secretion whereas acute intensive exercise or prolonged periods of exhausting exercise might decrease it, explaining the higher rate of mucosal infections observed in athletes (81). Here, our protocol of acute exercise did not induce significant changes in IgA concentration in SMGs or in the intestinal compartment, however, exercised rats had a lower concentration of salivary IgM, which has been described by others to be both decreased and increased after intensive exercise (12, 82). It must be taken into account that IgM is synthetized more quickly than IgA (83) and then it could be faster affected by an acute exercise. Moreover, it is important to clarify that whereas most of the published articles assessed changes in salivary secretory IgA, here we quantified the total concentration of IgA and IgM in submaxillary salivary gland homogenates, which may indicate changes in the ability to produce these antibodies, but not in the transcytosis and exocytosis processes. In further studies, we will ascertain the impact of acute exercise on salivary IgA and IgM concentrations, as well as how it affects their synthesis, exocytosis and transcytosis. Although the cocoa fiber diet, on its own, prevented this decrease in salivary IgM, the cocoa-enriched diet decreased it, both in the control and in exercised animals. These results are in line with results reported in healthy non-exercised rats (32). Moreover, in agreement with previous studies (36), the cocoa-enriched diet decreased the IgA concentration in SMGs. Therefore, cocoa diet, mainly due to bioactive compounds other than fiber, attenuated salivary Ig content. With regards to the intestinal compartment, animals from the CF and C10 groups had a lower concentration of IgA in GW after running.

In addition, to shed some light on the influence on mucosal immunity, we have studied the lymphocyte composition of two compartments of the gut-associated lymphoid tissue (GALT): the PPs and the MLNs. The GALT is the largest lymphoid tissue in the body and comprises the organized or inductor GALT, which includes PPs, MLNs and isolated lymphoid follicles, and the diffuse or effector GALT, which is formed by scattered intraepithelial (IEL) and lamina propria (LPL) lymphocytes. IgA-secreting plasma cells are mainly generated in PPs (84). It has been described that intense exercise, due to adrenergic mechanisms, induces a mobilization of splenic lymphocytes, mainly T cells, into secondary lymphoid organs such as PPs (6). In the current study, although we did not quantify the total number of cells, we did not observe any exercise-induced substantial change in the proportion of T and B cells in PPs, although it modified the proportion of the Tγδ cell subsets, favoring the presence of the Tγδ CD8αβ cells over that of Tγδ CD8αα cells in PPs. Tγδ CD8αβ cells have cytotoxic potential and secrete inflammatory cytokines, such as IFN-γ and TNF-α, that could contribute to the exercise-induced alterations in gut permeability that have been reported in other studies (37). On the other hand, the cocoa-enriched diet prevented the changes in Tγδ cell subsets in PPs, although it increased, as already described (85), the total proportion of Tγδ cells. These changes are not attributed to cocoa fiber content and may be due to other bioactive compounds such as polyphenols and methylxanthines. Nevertheless, cocoa diet, as already reported (85) increased the relative proportion of PPs Tc cells at the expense of Th cells, which could be partially associated with its fiber content.

The MLNs, together with the PPs, play an important role in initiating local immune responses in the gut (86). We found that, in this lymphoid organ, acute exercise induced an increase in NKT and Th cell proportions while it decreased that of Tc cells. These results agree with the reported higher Th/Tc cell ratio in MLNs of rats submitted to a 5-week intensive training (37). The results obtained here demonstrated that a single bout of exercise is able to change MLNLs proportions similarly to a 5-week training, probably due to differential mobilization of Th and Tc cells, but, on the contrary, while a 5-week training decreased the proliferative ability of T cells, the acute exercise applied here did not modify it. To our knowledge, there is no more available evidence on the effect of exercise on MLNs cell composition. Other authors have focused on brachial, axillary and submandibular lymph nodes, among others, but the results are not clear enough to draw any consistent conclusion (87). Cocoa fiber consumption, but not cocoa-enriched diet, prevented the changes induced by acute exercise in Th cells, suggesting the role of cocoa polyphenols or methylxanthines in counteracting the effect of cocoa fiber. On the other hand, both cocoa and cocoa fiber diets increased the proportion of Tγδ cells and decreased that of NKT cells. In the case of cocoa fed control animals, the higher proportion of Tγδ cells can be attributed to an increased proportion of Tγδ CD8αα cells, in agreement with a previous study (34).

Apart from these changes in T cell subset proportions in MLNs, acute exercise also modified their functionality, increasing the secretion of TNF-α, which could be explained by the cortisol released that occurs immediately after intensive exercise (29, 88, 89). Unlike what we previously observed in chronically intensively trained rats (29, 37), acute exercise did not manage to modify IL-2, IFN-γ, IL-4, and IL-6 production. On the other hand, although most of the studies reported a lower TNF-α production immediately after intensive exercise, normally involving a prior intensive training, due to the release of cortisol and noradrenaline (10, 90, 91), other authors have found no changes (13) or even an increased TNF-α production (92). Here, both acute exercise and cocoa fiber-enriched diet increased TNF-α secretion by *in vitro* stimulated MLNLs, in disagreement with previous studies showing an inhibitory effect on this cytokine production by cocoa consumption (20). Lastly, although our model of acute intensive exercise did not modify the IFN-γ levels released by stimulated MLNLs, the decrease induced by cocoa in the secretion of this proinflammatory cytokine could be useful to attenuate the substantial increase observed after a chronic intensive running training (37).

## Conclusion

In conclusion, our results indicate that the intake of cocoa in the days prior to an intense bout of exercise can partially prevent the alterations induced by it. Some of the changes produced by the cocoa diet on the intestine can be attributed to its fiber content, but other changes denote interaction between the phenolic compounds and methylxanthines, which is worth studying depth in the future. Moreover, further research might ascertain whether a more intensive exercise or a sustained exercise can exacerbate the observed alterations, and assess the potential protective effect of cocoa fiber in an even more impaired immune system.

## Data Availability Statement

The raw data supporting the conclusions of this article will be made available by the authors, without undue reservation.

## Ethics Statement

The animal study was reviewed and approved by Ethical Committee for Animal Experimentation of the University of Barcelona.

## Author Contributions

Under the supervision of MM-C and FP-C, PR-I completed these studies as part of her Ph.D. thesis. PR-I, MM-C, and FP-C contributed to the conception and design of the project. PR-I, MM-C, MR-L, AF, and MC-B performed the experimentation. PR-I conducted the literature search, the acquisition, analysis of data, and wrote the first version of the manuscript. All authors contributed to the revision of the manuscript, read, and approved the final work.

## Conflict of Interest

The authors declare that the research was conducted in the absence of any commercial or financial relationships that could be construed as a potential conflict of interest.

## Publisher’s Note

All claims expressed in this article are solely those of the authors and do not necessarily represent those of their affiliated organizations, or those of the publisher, the editors and the reviewers. Any product that may be evaluated in this article, or claim that may be made by its manufacturer, is not guaranteed or endorsed by the publisher.
